# The Relation Between War, Starvation, and Fertility Ideals in Sub-Saharan Africa: A Life History Perspective

**DOI:** 10.1177/14747049241274622

**Published:** 2024-10-11

**Authors:** Matthias Borgstede, Annette Scheunpflug

**Affiliations:** 1 14310 University of Bamberg, Bamberg, Germany

**Keywords:** life history theory, predictive adaptive response, personal fertility ideals, human development

## Abstract

In this article, we examine the relations between extreme environmental harshness during childhood and personal fertility ideals in African students. The study is informed by biological models of predictive adaptive responses (PAR) for individual reproductive schedules in the context of life history theory (LHT). Following theoretical models of external and internal environmental cues, we tested whether war and starvation during childhood differentially predict African students’ personal fertility ideals in terms of their desired number of children and their desired age of first parenthood. The data were collected in eight different countries from sub-Saharan Africa with an overall sample size of *N *= 392. Standardized effect estimates were obtained using a Bayesian approach. The results suggest that war and starvation are predictive of the desired number of children, but not of the desired age of first parenthood. Moreover, the effect estimates varied considerably between females and males, indicating possible interactions between the two independent variables depending on the students’ sex. Furthermore, we found a small negative correlation between the desired number of children and the desired age of first parenthood, providing only weak support for a clustering of the two variables on a slow–fast continuum. The results are discussed in light of current models of individual life histories in humans.

## Introduction

There is considerable empirical evidence that environmental harshness experienced early in life may affect adult behavior that is related to health, survival, and reproduction (cf. Chisholm et al., 2005; Del Giudice, 2014; Ellis et al., 2022; Sheppard et al., 2016; Webster et al., 2014). Evolutionary explanations of such findings are often inspired by life history theory (LHT). The common narrative states that LHT predicts individuals to adjust their life histories on a so-called fast–slow continuum. Within this line of theorizing, a fast life history is characterized by early maturation, high reproductive rate, and high mortality, whereas a slow life history is said to consist in late maturation, low reproductive rate, and low mortality (e.g., Jonason et al., 2010; Kubinski et al., 2017; Leiby & Madsen, 2017; Mittal & Griskevicius, 2014; Neel et al., 2016). Additionally, in evolutionary psychology, fast and slow life histories are often conceptualized with regard to various behavioral and psychological traits, such as parental care, mating behavior, sexual preferences, risk-taking, self-control, and even religious beliefs (Figueredo et al., 2005; Figueredo et al., 2007). There is a growing amount of research investigating how these traits may be shaped by individual experiences, suggesting that environmental harshness during childhood accelerates individual life histories, thereby favoring a tendency to start reproduction at a young age and produce a high number of offspring (cf. Nettle & Frankenhuis, 2020).

However, psychological applications of LHT have been repeatedly questioned on conceptual, theoretical, and empirical grounds (cf. Galipaud & Kokko, 2020; Sear, 2020; Stearns & Rodrigues, 2020). Nettle and Frankenhuis (2020) even propose that the biological and psychological versions of LHT are different (although related) research programs each consisting of their own conceptual frameworks, theoretical assumptions, and empirical paradigms. In biology, LHT is understood as a general framework to construct models of evolutionary trade-offs that explain how environmental and demographic conditions shape the solution of these trade-offs for different populations or species. Within biology, life history variables are unambiguously defined as reproduction, growth, and survival. The focus lies on formal models that make specific predictions for how these life history variables are affected by evolutionary trade-offs in different species or different populations. For example, the fundamental trade-off between present and future reproduction may be resolved very differently by various species depending on their environmental conditions. Furthermore, different solutions of evolutionary trade-offs do not necessarily result in a fast–slow continuum (cf. Dinh et al., 2022).

Nevertheless, the growing amount of empirical evidence on life history related traits suggests that evolutionary psychology may contribute substantially to the understanding of life history formation, especially with regard to proximate mechanisms and individual-level adaptations. However, care must be taken when concepts from LHT are applied in the social sciences. To avoid confusion regarding the different terminologies used in the biological and the psychological versions of LHT, in this article, we use the term “life history variables” only when we refer to the actual biological components of life histories, namely, reproduction, growth, and survival. For all other variables that might affect life histories (e.g., those involved in possible proximate mechanisms), we use the term “life history related variables.” Furthermore, we will not adopt the conceptual framework of a fast–slow continuum since the clustering of different physiological and behavioral traits in humans (a so-called pace-of-life syndrome, POLS) is only partly supported by empirical evidence (see Mathot & Frankenhuis, 2018 for an overview).

LHT itself says little about individual differences. Individual-level predictions require specific models that account for phenotypic plasticity within individuals (cf. Marty et al., 2011). These models usually distinguish between two possible mechanisms of how early-life experiences may shape future reproduction. Both approaches refer to the evolution of a *predictive adaptive response* (PAR; Gluckman et al., 2005). The first line of reasoning focuses on external factors, proposing that early-life experiences serve as statistical predictors of the future environment (cf. Bateson et al., 2004; Chisholm, 1999). The second approach incorporates internal factors, proposing that early-life experiences do not predict the future state of the environment, but rather the future state of the developing individual (cf. Epel et al., 2006; Selman et al., 2012; Wells, 2012). A possible external PAR might involve the signaling of a high extrinsic mortality rate during childhood (e.g., by means of experienced stress due to physical threat), to which the individual may respond with a corresponding reproductive schedule by producing more offspring and starting reproduction earlier. In contrast, an internal PAR would involve that the childhood environment affects the state of the individual. For example, bad nutrition during childhood may predict health impairments in adulthood, which may result in individuals to start reproduction at an earlier age. Consequently, internal predictive adaptive responses (PARs) are not responses to environmental harshness as such, but to its consequences on the individual itself. External and internal PARs may be effective simultaneously. Moreover, internal PARs may be more effective in the presence of cues of external harshness (Chang et al., 2019). In addition, the effects of PARs on individual reproductive schedules may be counterbalanced by other mechanisms that limit an individual's energy budget. Most importantly, malnutrition may have a direct effect on maturation and thus result in individuals starting to reproduce later in life. Therefore, it is difficult to derive predictions about actual reproductive schedules, especially with regard to the age of first reproduction (cf. Malani et al., 2023).

In humans, all of the abovementioned mechanisms seem possible and appear to be consistent with empirical observations (Chang et al., 2019; Chua et al., 2017; Hartman et al., 2017). However, the current research on PARs in human life histories appears to be mainly concerned with indicators of reproductive timing, such as age of menarche or age at first birth, or psychological traits that are not directly associated with fertility, but rather with psychological or behavioral factors that are thought to be life history-related, such as risk-taking behavior or time preference. In contrast, the effects of PARs on overall reproduction are far less studied (Coall et al., 2016). The same holds for psychological variables involved in proximate mechanisms that might affect overall reproduction in humans, such as fertility intentions or personal fertility ideals (but see Nettle et al., 2010). While fertility intentions designate concrete plans of reproductive behavior, they may be affected by personal ideals about parenthood (cf. Philipov & Bernardi, 2012). Personal fertility ideals are usually assessed by asking a person about the overall number of children they would like to have over the course of their lives. Such personal ideals usually form during development and persist into adulthood. Fertility ideals have been characterized as cognitive schemas that may affect fertility decisions directly (i.e., without deliberate processing) and indirectly (i.e., through the process of deliberate intention formation; Bachrach & Morgan, 2013). Although the number of desired children tends to decline with age and may change after the first childbirth, personal fertility ideals have been shown to be more persistent than fertility intentions and may thus serve as rough indicators for individual variation in expected reproductive behavior (cf. Heiland et al., 2008).

Furthermore, psychological studies on the effects of early-life experiences on individual life histories have largely focused on a very limited range of populations (Sear, 2020). Although the evolutionary framework provided by LHT should be universally applicable, much research still relies on data from western, educated, industrialized, rich, and democratic societies (WEIRD; Henrich et al., 2010). These societies usually have a comparably low variance in environmental conditions when compared to countries from the global south. In other words, WEIRD samples are not typically exposed to very harsh environmental conditions. Only few studies have investigated the effects of severe threat, such as it is experienced during periods of war, or prolonging malnutrition, such as it is experienced during periods of starvation (see, however, Međedović, 2019 and Lynch et al., 2018). Since the effects of such extreme environmental cues should be stronger than the effects of the variables that are usually studied in western populations (e.g., father absence or below average income), more empirical evidence from non-WEIRD populations might provide valuable information about the scope of evolutionary approaches to the development of human life histories (cf. Sear et al., 2019). Finally, most works on the effects of early life experiences on individual life histories focus on female reproductive schedules. However, since selection on reproductive strategies may vary between males and females, it is worthwhile exploring differential effects for males and females, separately (cf. Galipaud & Kokko, 2020).

In this article, we study the effects of a harsh environment during childhood on personal fertility ideals in a sample from eight different African countries, which are all considered to be of alarming or high fragility by the Fund for Peace (Fund for Peace, 2022). Environmental harshness is assessed in terms of war and starvation during childhood. Although war and starvation are not independent of one another (cf. Stewart, 2002), they roughly align with the two mechanisms of external and internal PARs. Empirically, war and starvation have been shown to be associated with parental care on the individual level, which is closely related to human reproductive behavior (Quinlan, 2007). Furthermore, both factors can be linked to an increase in extrinsic mortality (i.e., mortality risks that are independent of the individuals’ behavior). Although its theoretical role for the development of individual adaptive behavior seems to be more complex than often thought (De Vries et al., 2023), extrinsic mortality has been shown to be associated with life history-related variables (e.g., Chisholm, 1999). As an indicator of individual reproductive schedules, we assess personal fertility ideals, as expressed in the stated number of desired children and the stated ideal age of first parenthood. We will investigate whether a harsh environment during childhood predicts personal fertility ideals in adolescents and young adults and whether the internal and external cues are independent of one another. As a general heuristic, we expect a harsh environment during childhood to be associated with a higher number of desired children and an earlier ideal age of first parenthood. However, we make no specific predictions regarding the clustering of the two life history-related variables.

## Methods

The data were collected between September 2019 and March 2020^1^ by the participants of an international masters program, who worked as teachers in eight different African countries (Burundi, Cameroon, Democratic Republic of Congo, Liberia, Rwanda, Tanzania, Uganda, Zambia), all of which have been rated between “high warning” and “very high alert” on the Fragile States Index for the last 20 years (Fund for Peace, 2022).

Questionnaires were delivered to students of secondary schools (which usually starts after 6 years of primary school) at the workplaces of the masters program participants. The questionnaires were reviewed and approved by the headmasters of all schools to ensure that they complied with the local regulations for data collection in schools. Moreover, all students and all legal representatives of the students who were younger than 18 years at the time of the data collection were informed about the content and the aims of the study prior to the data acquisition. Parents were given the opportunity to object to their children's participation either in written form or verbally (the latter was necessary because some parents had a low level of literacy). Students were asked to fill out the questionnaire during classes. All respondents were informed about their right to deny being part of the study and of full anonymity. The questionnaires were first constructed in English, and then translated into French. An independent back-translation was performed to ensure the semantic equivalence of the two questionnaire versions. Since the level of literacy among the participants varied, both versions of the questionnaires were double-checked by the teachers of the participants to ensure that all students would be able to understand the questions. Moreover, students had the opportunity to ask questions regarding the questionnaire during data collection.

Personal fertility ideals were assessed by means of two numerical ratings. The number of desired children was assessed by two items, one asking for the number of desired sons and one for the number of desired daughters (“How many sons/daughters do you wish to have over the whole span of your life?”). The optimal age of first parenthood was assessed using a single item (“In your opinion, what is the best age to become a parent for the first time?”). Two retrospective self-assessment items were presented to assess external and internal cues of environmental harshness, one for war experience (“During your childhood, have you ever experienced war?”) and one for starvation (“During your childhood, have you ever had so little food that you were hungry for several days in a row?”). To account for possible confounding variables, the questionnaire contained questions about the participants’ gender (“male” vs. “female”), their current age in years, and whether they were already parents at the time of the study (“Do you have any children of your own?”). As additional control variables, family wealth and the current safety of the participants’ neighborhood were assessed on a seven-point scale (“How would you rate your family's wealth on a scale from 1 (very poor) to 7 (very rich)?” and “How would you rate the safety of your neighborhood on a scale from 1 (very dangerous) to 7 (very safe)?”).

The data were analyzed in terms of descriptive and multivariate statistics using the free statistics environment, R, version 4.0.3 (R Core Team, 2020). All statistical estimates were obtained using a Bayesian approach with weakly informative priors, as implemented in the R-package rstanarm, version 2.32.1 (Goodrich et al., 2024). For all analyses, the median of the respective posterior distribution is reported as a point estimate, alongside a 90% credible interval. Each credible interval is estimated directly from the posterior distribution and indicates an area around the estimated parameter that contains the true parameter with a probability of 0.9. In particular, we calculated pairwise Bayesian correlation coefficients for all variables that were included in the analysis, followed by a series of Bayesian multiple linear regression models to assess the multivariate relations between the variables of interest. In line with the Bayesian approach, we do not report *p*-values or levels of significance. Instead of testing population level hypotheses, we thereby focus on the estimation of effect sizes. This leaves the question of external validity to non-statistical generalization strategies, as proposed in Borgstede and Scholz (2021).

Although the data were hierarchically nested within countries, we did not include country as a clustering variable in the regression models. There are several reasons for this decision. First, country-level effects are likely to be highly unreliable due to the small number of countries and cases within countries (e.g., Stegmueller, 2013). Second, for 29 individuals, no information about the country is available, which would have introduced a considerable amount of missing values to the data set. Third, the independent variables (i.e., war and starvation) can be expected to be confounded with the country variable. Controlling for country would partialize out all country dependent effects on war and starvation. However, these effects are theoretically relevant for the formation of personal fertility ideals in the context of the current study. Therefore, including country as a clustering variable would negatively bias the parameter estimates of the independent variables.^2^

To facilitate the interpretation of the effect estimates, all non-binary variables were standardized prior to the analysis. The two dichotomous variables indicating war and starvation experiences were used to calculate dummy variables for “war experiences only,” “starvation experiences only,” and “war and starvation experiences,” with the reference category being “neither war nor starvation experiences.” This coding scheme was preferred over binary coding with an additional interaction term because the main interest of the study lies in the singular and combined effects of the two variables compared to a neutral condition, rather than the partitioning of main effects and interaction effects. Moreover, the applied coding scheme allows for a straightforward interpretation of the model parameters in the sense of unique and combined effects.

The multivariate analyses consisted of a series of Bayesian linear regression models that were fitted for each of the two dependent variables (desired number of children and desired age of first parenthood). The models were estimated for the whole sample, as well as for males and females, separately.^3^

## Results

The overall sample size was *N *= 392. Of the total sample, 289 subjects were English-speaking (73.7%), and 103 were French-speaking (26.3%). Overall, 195 subjects reported being male (49.7%); 191 indicated that they were female (48.7%); and six did not indicate their gender (1.5%). The mean age of the subjects in the original sample was 20.6 years (*SD *= 4.9 years). One individual who did not indicate their age and two individuals who were still children when the study took place (aged 12 years or younger) were excluded from the analysis. The high range in student age may be partly due to the high percentage of children who have experienced war (and, thus, were not able to attend school for at least some time). Apart from this, children may pause their school attendance because their family cannot afford the school fees or needs them to work and later resume their formal education. Furthermore, 45 individuals reported to have at least one child at the time of the study. Tables 1 and 2 summarize the main descriptives of the final sample with regard to the assessed variables.

**Table 1.** Descriptives of the Assessed Variables.

	Mean	SD	Min	Max	Missing
Age (years)	20.57	4.89	14	32	0
Wealth (1–7)	3.99	1.57	1	7	0
Safety (1–7)	4.71	1.78	1	7	0
Desired number of children (count)	4.48	1.66	0	12	1
Ideal age of first parenthood (years)	26.32	3.96	13	38	1

**Table 2.** Distribution of War and Starvation Experiences during Childhood over Countries.

	Total count	Neither war nor starvation	War only	Starvation only	War and starvation	Missing
Burundi	23	1	17	2	3	0
Cameroon	99	64	13	17	2	3
DR of Congo	61	19	27	4	10	1
Liberia	21	2	5	0	11	3
Rwanda	109	51	2	44	9	3
Tanzania	24	7	2	11	3	1
Uganda	7	1	4	0	2	0
Zambia	19	17	1	1	0	0
Unknown	26	4	2	0	19	1
Sum	389	166	73	79	59	12

The range of the dependent variables is exceptionally high in comparison to samples from developed countries, with a desired number of children between 0 and 12 and a desired age of first parenthood between 13 and 38. In all countries, a substantial proportion of the subjects had experienced war (between 5% and 87%) or starvation (between 5% and 58%) during their childhood. Overall, 29% of the subjects had experienced war and 30% starvation during their childhood. Furthermore, 14% of the subjects reported experiencing both war and starvation during their childhood. The number of observations varies considerably between the different countries (between 7 and 109). By far the largest proportion of students came from Rwanda, which might have a significant influence on the effect estimates. However, re-running the analyses without the Rwandan students did not change the overall pattern of the reported results.

Table 3 depicts the pairwise Bayesian correlation estimates between all assessed variables (note that for the binary variables, the reported Pearson correlation is identical with the Phi coefficient for binary associations). The table shows that there were small negative correlations between the independent variables (war and starvation). War was further positively correlated with desired number of children, but not with the ideal age of first parenthood. The current safety of the environment was negatively related to war. Starvation showed a positive correlation with age (indicating a possible cohort effect) and a negative correlation with wealth. Sex was positively correlated with age (indicating that the male students were, on average, older than the female students) and with ideal age of first parenthood (indicating that the male students preferred, on average, having their first child at a higher age than the female students). Finally, there was a moderate correlation between wealth and safety. All other correlations were very small, with 90% credible intervals, including zero.

**Table 3.** Pairwise Bayesian Correlations Between the Assessed Variables.

	1.	2.	3.	4.	5.	6.	7.
1. War	-						
2. Starvation	**0.12** (0.04, 0.2)	-					
3. Desired number of children	**0.11** (0.03, 0.19)	0.08 (0, 0.16)	-				
4. Ideal age of first parenthood	0.02 (−0.06, 0.11)	0.01 (−0.07, 0.1)	−0.09 (−0.17, 0)	-			
5. Sex (male)	0.02 (−0.07, 0.1)	0.07 (−0.01, 0.16)	0.03 (−0.06, 0.11)	**0.36** (0.29, 0.43)	-		
6. Age	0.09 (0, 0.17)	**0.23** (0.15, 0.3)	−0.04 (−0.12, 0.04)	0.08 (−0.01, 0.16)	**0.17** (0.09, 0.25)	-	
7. Wealth	−0.01 (−0.09, 0.07)	**−0.33** (−0.4, −0.25)	0.01 (−0.07, 0.1)	−0.02 (−0.1, 0.06)	0.02 (−0.06, 0.11)	**−0.15** (−0.23, −0.07)	-
8. Safety	**−0.16** (−0.24, −0.08)	−0.07 (−0.16, 0.01)	−0.01 (−0.1, 0.07)	0.04 (−0.05, 0.12)	0 (−0.08, 0.08)	**−0.17** (−0.25, −0.09)	**0.36** (0.29, 0.43)

*Note:* Pairwise associations (Pearson correlations), Bayesian estimates with 90% credible intervals.

The results of the Bayesian linear regression models are summarized in Tables 4 and 5. Table 4 presents the standardized effect estimates for the desired number of children. When the whole sample is used, both independent variables have a positive effect (however, with a considerable amount of estimation error, as indicated by the 90% credible intervals). When war and starvation occur together, the corresponding effect estimate from the complete sample is approximately equal to the sum of the effects of the two independent variables (with a 90% credible interval that does not contain zero). For the female sample, the joint effect of the two independent variables is approximately the same as in the complete sample (with a 90% credible interval that does not contain zero). However, the effect estimates for war only and starvation only differ from the estimates obtained from the complete sample. The effect estimate of war only is less than half as big as in the complete sample, and the effect estimate of starvation only is negative. However, the credible intervals for these effects are large and both contain zero. For the male sample, the effect estimates for war only and starvation only are both larger than in the complete sample (with a 90% credible interval not containing zero for starvation only). However, the estimate of the joint effect of war and starvation is smaller than in the complete sample (with a 90% credible interval not containing zero). The overall model fit appears low since the Bayesian *R*^2^ indicates only an amount of 4% explained variance in the dependent variable.

**Table 4.** Results of Bayesian Linear Regression Models Predicting Desired Number of Children.

	Desired number of children (complete sample)	Desired number of children (females only)	Desired number of children (males only)
*Predictors*	*Estimates*	*Estimates*	*Estimates*
(intercept)	−0.17 (−0.34, 0.00)	−0.08 (−0.28, 0.12)	−0.18 (−0.42, 0.07)
Sex	0.06 (−0.14, 0.26)		
Age	−0.06 (−0.17, 0.04)	−0.08 (−0.23, 0.07)	−0.08 (−0.23, 0.07)
Wealth	0.07 (−0.05, 0.17)	0.12 (−0.02, 0.26)	0.02 (−0.18, 0.21)
Safety	−0.05 (−0.16, 0.07)	0.06 (−0.07, 0.20)	−0.14 (−0.33, 0.06)
War only	0.21 (−0.06, 0.50)	0.08 (−0.27, 0.44)	0.34 (−0.09, 0.78)
Starvation only	0.19 (−0.08, 0.46)	−0.17 (−0.55, 0.19)	**0.48** (0.05, 0.90)
War and starvation	**0.41** (0.11, 0.73)	**0.46** (0.04, 0.88)	0.33 (−0.14, 0.79)
*N*	371	183	188
*R^2^* Bayes	0.040	0.094	0.073

*Note:* Bayesian standardized effect estimates with 90% credible intervals.

**Table 5.** Results of Bayesian Linear Regression Models Predicting Ideal Age of First Parenthood.

	Ideal age of first parenthood (complete sample)	Ideal age of first parenthood (females only)	Ideal age of first parenthood (males only)
*Predictors*	*Estimates*	*Estimates*	*Estimates*
(intercept)	**−0.34** (−0.52, −0.16)	**−0.41** (−0.59, −0.23)	**0.48** (0.24, 0.72)
Sex	**0.74** (0.54, 0.93)		
Age	0.01 (−0.09, 0.11)	0.02 (−0.13, 0.17)	0.02 (−0.13, 0.17)
Wealth	−0.07 (−0.18, 0.05)	−0.03 (−0.17, 0.11)	−0.13 (−0.31, 0.05)
Safety	0.07 (−0.04, 0.18)	0.01 (−0.11, 0.14)	0.15 (−0.03, 0.34)
War only	−0.07 (−0.33, 0.20)	0.06 (−0.26, 0.39)	−0.23 (−0.66, 0.20)
Starvation only	−0.21 (−0.47, 0.07)	0.01 (−0.35, 0.35)	−0.40 (−0.81, 0.01)
War and starvation	0.08 (−0.23, 0.38)	0.15 (−0.26, 0.54)	0.01 (−0.45, 0.46)
*N*	371	182	189
*R^2^* Bayes	0.155	0.034	0.058

*Note:* Bayesian standardized effect estimates with 90% credible intervals.

Table 5 presents the standardized effect estimates for the ideal age of first parenthood. In contrast to the desired number of children, the effect estimates for the joint effect of the independent variables obtained from the complete sample are close to zero (again, with a considerable amount of estimation error, as indicated by the 90% credible intervals). Moreover, the effect estimates of the effects of war only and starvation only are negative (however, with 90% credible intervals containing zero). The only effect estimate that clearly differs from zero (indicated by the 90% credible interval) refers to sex, with males reporting a higher ideal age of first parenthood than females. This effect is mirrored in the intercepts of the models obtained from the female and male samples, separately. For the female sample, the joint effect of the two independent variables is higher than in the complete sample (however, the 90% credible interval contains zero). The effect estimates for war only and starvation only are both slightly positive, but have a wide range of estimation error (with 90% credible intervals containing zero). For the male sample, the joint effect of the independent variables is again very small (with a 90% credible interval that contains zero). The effect estimates for war only and starvation only are both negative (however, the 90% credible intervals contain zero). The overall model fit is rather low for the male and female samples since the Bayesian *R*^2^ indicates only 3% to 6% explained variance in the dependent variable. For the complete sample, the overall model fit is better, with a Bayesian *R*^2^ indicating about 16% explained variance. The better fit of the complete sample model suggests that the best predictor of the ideal age of first parenthood is the students' sex, rather than any of the independent variables.

**Figure 1.**
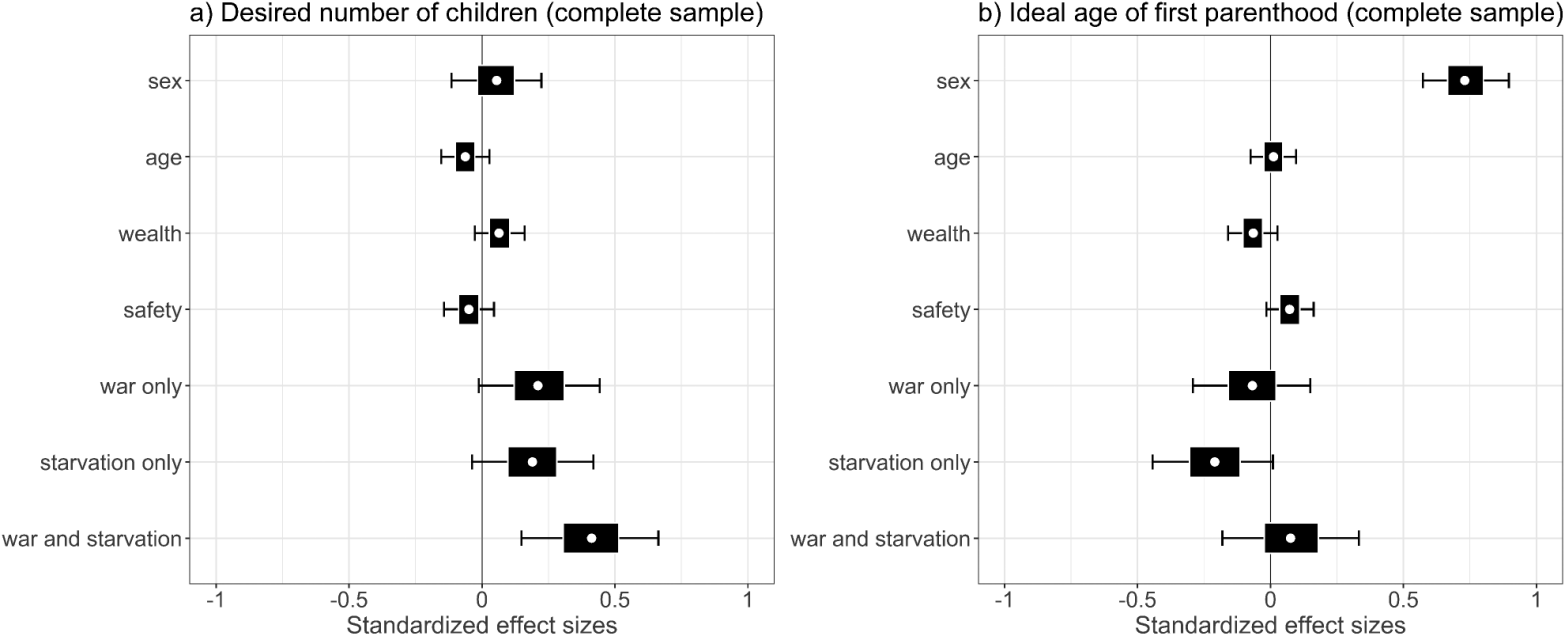
Effect size plots from Bayesian regression models for desired number of children and ideal age of first parenthood. White circles indicate the median; the boxes indicate the inter-quartile-range; and the whiskers indicate the 5th and 95th percentiles of the posterior predictive distributions for each parameter.

The main results of the Bayesian regression analysis are illustrated in Figure 1. The figure depicts the standardized effect size estimates obtained from the complete samples for the desired number of children (panel a) and the ideal age of first parenthood (panel b). The effect size estimates are presented as box-and-whiskers-plots, with circles being the median, box margins being the inter-quartile-range, and whiskers indicating the 5% and 95% quantiles of the posterior predictive distribution. Figure 1 shows that the effects of war and starvation appear to be additive with the joint effect of war and starvation clearly differing from zero, and that the only estimate that substantially differs from zero for the ideal age of first parenthood is the students’ sex.

## Discussion

The aim of this study was to investigate the effects of environmental harshness on fertility ideals using a sample from eight different countries from sub-Saharan Africa. The study was informed by the general evolutionary framework of LHT and by the more specific concepts of internal and external PARs. Environmental harshness during childhood was assessed in terms of past experiences of war and starvation. Fertility ideals were assessed in terms of the desired number of children and the desired age of first reproduction. In line with theoretical models, we predicted that both indicators of environmental harshness would be associated with a higher desired number of children and a lower desired age of first reproduction. We did not, however, make specific predictions about the clustering of the two life history-related variables (desired number of children and desired age of first reproduction) on a fast–slow continuum. The results only partly confirmed these predictions, with war and starvation during childhood being positive predictors of a higher desired number of children and a combined effect that indicates an independent contribution of the two factors to the joint effect. However, when analyzed separately by students’ sex, starvation had a negative effect on the desired number of children for females and a positive effect for males, suggesting possible interactions between students’ sex and the independent variables. With regard to the ideal age of first parenthood, no substantive effects of environmental harshness were found. Finally, desired number of children and ideal age of first parenthood showed only a small negative correlation, thereby providing only weak support for a clustering of personal fertility ideals along a fast–slow continuum.

Regarding the desired number of children, two different interpretations of the data are possible. First, the effect estimates from the complete sample suggest that both war and starvation during childhood may increase the desired number of children via two independent mechanisms (external and internal PARs) to produce an approximately additive joint effect. However, the separate analyses for females and males indicate that the situation may be more complex with different effects depending on sex. Therefore, it would be equally plausible that war only and starvation only have a stronger predictive value for the future prospects of males than for females (e.g., because males are more likely to be actively involved in violent conflicts once they are grown up), causing stronger effects on the desired number of children in males than in females, which might result in a ceiling effect when both factors occur simultaneously. Meanwhile, for females, war might be less predictive of the individuals’ future state (cf. Nettle et al., 2013), such that an external PAR might not have evolved for females at all. For starvation, the negative effect found in the female sample might actually be the result of delayed maturation due to malnutrition — a mechanism that acts in the opposite direction when compared to a possibly extant internal PAR and might be more effective if starvation occurs on its own, but counterbalanced when a female child experiences starvation in the context of war. The smaller effect of war on the desired number of children for female students might also reflect sex-specific influences of education on fertility ideals. Especially in developing countries, an increase in womens' education is negatively related to their desired number of children (Jejeebhoy, 1995), which might explain why the estimated war effect on the desired number of children is much smaller for female students than for male students in the current study.

The results found for the desired age of first parenthood show neither conclusive evidence for internal or internal PARs nor for a possible delayed maturation effect. Instead, the only substantive predictor of the ideal age of first parenthood appears to be the students’ sex, with males preferring to start reproduction at an older age compared to females. Only in the male sample, there were small negative effects of war and starvation on the ideal age of first parenthood (however, with considerable prediction error). In the complete sample and the female sample, neither war or starvation nor the combination of the two showed substantive effects on the dependent variable. One possible reason for these null results might be that personal fertility ideals are not as reliable for reproductive timing as they are for overall reproductive effort (cf. Heiland et al., 2008). Alternatively, war and starvation might actually only affect overall reproduction, but not reproductive timing. Since the negative correlation between the two is only small, such differential effects appear to be possible. However, human reproductive behavior is complex and may vary depending on the particular demography of a population. In fact, war and starvation are directly related to mortality risks that do not only affect individual ideals, but also migration patterns, labor markets, and societal norms, all of which may interact with individual reproductive behavior. Many of these socio-cultural effects may be interpreted as proximate mechanisms that realize an evolved behavioral response. For example, increased fertility as a response to child mortality is sometimes explained by parents’ tendencies to replace deceased children or to insure themselves against future child loss (LeGrand et al., 2003; Rutayisire et al., 2013), which would be perfectly in line with evolutionary theory. However, some socio-cultural mechanisms might actually be independent of the kind of biological trade-offs addressed in LHT (cf. Skirbekk, 2022). A complete understanding probably requires dedicated formal models that allow for specific predictions and more studies from diverse cultural backgrounds (Galipaud & Kokko, 2020).

Various aspects in the design of the study limit the implications of the results. The use of retrospective self-reports may seem less reliable than objective observational data. However, although there certainly is some room for interpretation, the independent variables are rather definite. In other words, people will most likely know whether they experienced war or starvation in the past. Furthermore, one might argue that fertility ideals are only imperfect indicators of individual life history variables, such as actual reproductive onset or the actual number of offspring over the lifetime of an individual. However, much of human reproductive behavior is likely mediated by psychological processes, such as intention formation. Moreover, there is convincing theoretical and empirical evidence that reproductive behavior is in fact related to personal fertility ideals (Miller, 1994, 2012). Therefore, although the predicted effects will most likely be smaller than with regard to actual reproductive schedules, personal fertility ideals may still be informative to a certain degree. Another limiting factor might be the sampling strategy employed in the study. Using only data from children who attend secondary school might have excluded those individuals who suffer most from violent conflict or low socio-economic status. In fact, in the eight countries that were examined in this study, the enrollment ratio in secondary schools varied between 43% and 70% with an average rate of 55.5% (The World Bank, 2024). Consequently, it might be possible that the true population effects of the independent variables are in fact larger than estimated from the obtained data. However, the average gender parity index for secondary school enrollment lies between 0.99 and 1, indicating that there was no sex-specific bias due to the sampling procedure (The World Bank, 2024). Finally, the scope of the study is limited by the correlational design, which makes it difficult to interpret the statistical effects in terms of causal mechanisms. Nevertheless, controlling for various background variables, including the current safety of the environment, suggests that the effects actually reflect differences conditional on early childhood experiences, and not the effects of the current environment.

A strength of this study is that the sample includes a substantial amount of people who experienced extreme levels of early stress due to war and starvation. Studies in more developed countries rarely capture such extreme experiences. Instead, much research on individual life histories in humans focuses on family relations, such as father absence or attachment styles (cf. Chisholm et al., 1993). While impaired family relations may in fact be relevant for future reproductive behavior (Webster et al., 2014), the specific predictions of internal and external PARs might be more suitably tested with regard to environmental variation with immediate evolutionary relevance. For example, experiencing war implies the chronic threat of being severely injured, being separated from one's family, or even being killed. Similarly, starvation is an immediate physical threat and directly affects the somatic system and child development in particular (Dipasquale et al., 2020; Rizzo et al., 1997).

Taken together, this study provides new evidence that human reproductive behavior might be sensitive to childhood experiences due to evolved predictive adaptive responses to environmental cues, as well as energetic constraints due to malnutrition. The results imply that personal fertility ideals might be biased toward a higher number of desired children when an individual experiences a strong environmental cue, such as war or starvation. These personal fertility ideals may further affect actual reproductive behavior in humans. However, we found no corresponding effect for desired age of first parenthood or for a clustering of the two life history-related variables. These results suggest that individual-level life history adaptations may be rather complex and do not necessarily reduce to a fast–slow continuum.

## Notes

1. The data were collected before contact restrictions due to COVID became effective in the respective schools.

2. Re-running the analyses with country included as either a random or fixed effect did in fact reduce the effect estimates.

3. As a robustness check, the models were fitted with and without the control variables, which hardly changed the effect estimates. Therefore, only the results from the models with controls are reported. Note that we did not include the number of own kids as a control variable since actual parenthood might be a result, rather than a cause, of fertility ideals. Re-running the analysis without the 45 students who were parents produced essentially the same results for desired number of children, but increased effect estimates for ideal age of first parenthood. Since the ideal age of first parenthood is known to be correlated with the actual age of first parenthood, the latter effect most probably is the result of a selection bias, rather than substantive.
